# Nimesulide-Induced Fixed Drug Eruption Followed by Etoricoxib-Induced Fixed Drug Eruption: An Unusual Case Report and Review of the Literature

**DOI:** 10.3390/jcm13061583

**Published:** 2024-03-10

**Authors:** Michael Makris, Niki Papapostolou, Ioannis-Alexios Koumprentziotis, Georgia Pappa, Alexander C. Katoulis

**Affiliations:** 1 Allergy Unit “D. Kalogeromitros”, 2nd Department of Dermatology and Venereology, Medical School, “Attikon” University Hospital, National and Kapodistrian University of Athens, 12462 Athens, Greece; mmakris.allergy@gmail.com (M.M.); giannhskmpr@gmail.com (I.-A.K.); 2 2nd Department of Dermatology and Venereology, Medical School, “Attikon” University Hospital, National and Kapodistrian University of Athens, 12462 Athens, Greece; alexanderkatoulis@yahoo.co.uk

**Keywords:** fixed drug eruption (FDE), non-steroidal anti-inflammatory drugs (NSAIDs), nimesulide, etoricoxib, cross-reactivity, hypersensitivity reaction, cutaneous drug reaction

## Abstract

Fixed drug eruption (FDE) is a well-recognized, non-immediate, drug hypersensitivity reaction, often attributed to the use of various medications, most commonly non-steroidal anti-inflammatory drugs (NSAIDs) and antibiotics. Cross-reactivity between related NSAIDs in FDE has been reported, but among chemically unrelated NSAIDs, is rare. Herein, we present a rare well-documented case where a patient initially displayed tolerance to etoricoxib after experiencing a nimesulide-induced FDE. Subsequently, the patient developed an etoricoxib-induced FDE, accompanied by the development of bullous lesions. This case report and the literature review on comparable FDE occurrences shed light on the intricate nature of FDEs, suggesting the possibility of cross-reactivity between chemically related and unrelated NSAIDs or the emergence of new drug-specific T cells without cross-reactivity after multiple exposures to a drug in a susceptible patient. Our case underscores the importance of increased awareness and vigilance among both physicians and patients in the realm of personalized medicine. Further research is needed to unravel the intricate mechanisms behind these drug eruptions, improve diagnostic approaches, and enhance patient care.

## 1. Introduction

Fixed drug eruption (FDE) is a relatively common cutaneous drug reaction accounting for up to 10% of all skin drug reactions and comes after morbilliform and urticarial drug reactions. FDE can be attributed to a variety of medications, with the most common culprit drugs being non-steroidal anti-inflammatory drugs (NSAIDs) and antibiotics. Sedatives, anticonvulsants, antihypertensives, and several other medications have been identified as less common causes of FDE [1,2,3,4,5].

Pathogenetically, FDE is a delayed type of IVc hypersensitivity reaction which is considered to cause damage in the skin basal layer with consequential activation of resident CD8+ T cells. This response leads to the release of various mediators, such as interferon (IFN)-γ, and the recruitment of other immune cells causing damage to keratinocytes and melanocytes and the eventual appearance of the characteristic lesions [4,6,7,8].

Clinically, it is characterized by the recurrence of single or multiple cutaneous and/or mucosal lesions that typically consist of well-defined round or oval, egg-sized patches of dusky violaceous or brownish color after the use of the causative agent. Occasionally, blisters and/or bullae can develop in the violaceous discoid erythema. Based on the lesional morphology, locations affected, and distribution, FDE can be further subcategorized, with some of the main clinical variants being pigmented, bullous, non-pigmented, generalized fixed drug eruption (GFDE), and generalized bullous fixed drug eruption (GBFDE) [4,5,6]. Most of the FDE variants run a generally benign course and are not life-threatening, with the rare exception of GBFDE, which has been reported as a rare cause of mortality. After resolution, it is common for residual hyperpigmentation to occur in the involved sites lasting weeks to months. The lesions can reappear at the same or different sites after the re-administration of the causative agent [4,5,7]. While not very common, cross-reactivity causing various reactions is known to occur between NSAIDs belonging to the same chemical family and thus sharing similar structures. Because of that, a common practice after identifying the offending agent is to avoid re-exposure to those medications and those that structurally resemble them in order to abstain from recurrences [4,7]. However, cases of immunological cross-reactivity among chemically unrelated NSAIDs have seldom been documented in the existing literature but can be the cause of a diagnostic pitfall and affect the therapeutic approach [9]. NSAIDs are among the most commonly used medications in the modern era and physicians should be aware of possible reactions caused due to cross-reactivity between such medications. Herein, we present the case of a male patient who initially manifested an FDE attributed to nimesulide. After a year during which the patient took 3–4 courses of etoricoxib with no adverse reactions, an additional course of etoricoxib precipitated the onset of an FDE. We also provide a comprehensive literature review of studies and cases presenting patients with FDEs that occurred due to cross-reactivity between both chemically related and unrelated groups of NSAIDs in order to add to our knowledge of such rare reactions.

## 2. Case Description

On June 2021, a 68-year-old Caucasian male presented to the Emergency Department of the Second Department of Dermatology and Venereology in “Attikon” General University Hospital, Athens, Greece, due to a week’s onset of round to oval, dusky red to brown/black well-demarcated macules on his upper back and oral mucosa, almost 8 h after taking a nimesulide 100 mg tablet (Mesulid^®^, Boehinger Ingelheim AE, Athens, Greece) for sciatica. The patient did not use any other medications except for latanoprost eye drops for glaucoma and was otherwise healthy. The patient reported similar lesions of milder severity that had appeared in the last 12 months after the use of nimesulide 100 mg (Mesulid^®^, Vianex AE, Athens, Greece) but were not evaluated by a physician.

**Figure 1:**
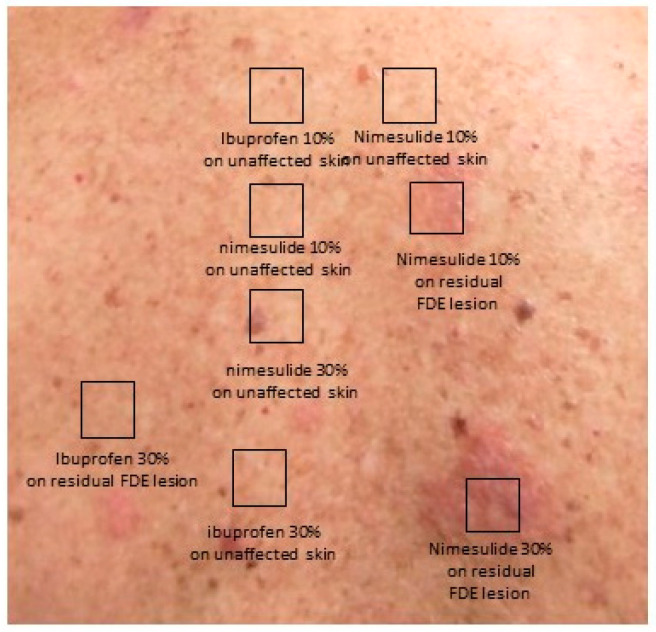
Positive patch tests (weight/volume preparations in petrolatum) to nimesulide 10% on previous FDE lesion and nimesulide 30% on previous FDE lesion; negative results to nimesulide 10% on unaffected skin, nimesulide 30% on unaffected skin, ibuprofen 30% on previous FDE lesion, ibuprofen 10% on unaffected skin, and ibuprofen 30% on unaffected skin.

The physical examination revealed three round to oval, dusky red-brown/ black well-demarcated macules on his back and one in his oral mucosa. Based on the clinical presentation, a nimesulide-induced FDE was suspected. Although a skin biopsy was recommended, the patient did not consent. The diagnostic work-up with patch tests was performed two months after the resolution of the rash at the sites of previous lesions, with normal skin used as a control, following international guidelines [9]. Patch tests were conducted using the suspected culprit drug, nimesulide 100 mg (Mesulid^®^, Vianex AE) and with ibuprofen 600 mg (Algofren^®^, Intermed ABEE, Athens, Greece) as an alternative tested NSAID. Preparations containing 10% and 30% weight/volume in petrolatum were applied to the previous residual pigmented lesions and normal skin. The 48 and 72 h readings revealed a positive reaction to nimesulide 10% and 30% on the residual hyperpigmentation areas, while all other performed tests were negative (Figure 1). Given that the patient had used etoricoxib 90 mg (Arcoxia^®^, Vianex AE) for musculoskeletal pain without experiencing any adverse reactions after the initial nimesulide-attributed reaction, no in vivo and/or in vitro tests were conducted for etoricoxib. Consequently, the patient was advised to abstain from using nimesulide and was recommended to use ibuprofen, based on the negativity of patch tests, and etoricoxib which he had tolerated after the initial nimesulide-induced FDE. Thus, over the course of the following year, the patient received 3–4 courses of etoricoxib 90 mg (Arcoxia^®^, Vianex AE), each lasting 3–5 days, all of which were well-tolerated. He was also prescribed meloxicam for 7 days by his orthopedic, which also was well tolerated.

**Figure 2:**
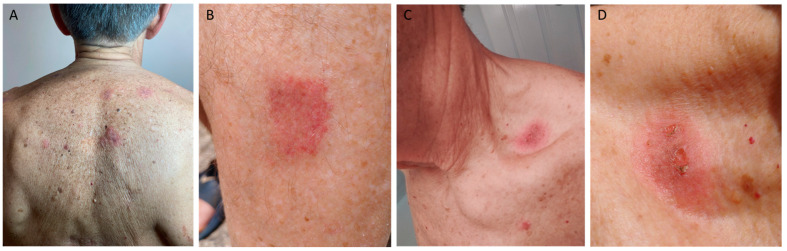
(**A**–**D**) Fixed drug eruption typical skin lesions after etoricoxib intake on the back (**A**), left tibia (**B**), and lateral neck with ruptured bullae (**C**,**D**).

In November 2022, the patient underwent hip replacement surgery and was instructed to use etoricoxib 90 mg and paracetamol 500 mg up to 3 times daily for his post-surgical recovery period. Three days after the surgery and the use of the drugs, the patient experienced discomfort in his oral cavity, contralateral to the lesion that was caused by nimesulide 1 year before. Thus, he was evaluated by his dentist and no new lesions were observed in the oral mucosa. Additionally, no new cutaneous lesions were detected, and the old lesions had disappeared, leaving minimum residual post-inflammatory hyperpigmentation. Because of high clinical suspicion and the previous history of FDE, the patient was instructed to continue only with paracetamol and discontinue etoricoxib until further evaluation was performed. Based on the nature of the reaction (type IVc delayed drug hypersensitivity reaction according to the Gell and Coombs modified classification), a Lymphocyte Transformation Test (LTT) was ordered for ibuprofen, etoricoxib, and nimesulide, all of which yielded negative results for the substances with low Stimulation Indexes (SI: 1.01, 1.12 and 1.20, respectively). Oral provocation tests (OPT) with one therapeutic dose for each drug were performed, using ibuprofen and, on a separate day, etoricoxib, both being negative. Six months later, the patient received etoricoxib again for the first time after the OPT and reported a feeling of generalized pruritus a few hours after taking the drug for his sciatica but without the occurrence of any new exanthem or enanthem. The next day, etoricoxib was used again and shortly after, multiple oval brownish macules gradually appeared in multiple locations. After assessment, multiple macules were detected on the back, two lesions on the lateral neck, three on the right forearm, one on the left tibia, and three on the face (Figure 2A–D). Two of the macules detected on the back of the patient reappeared at the same sites where they had appeared during the previous nimesulide-induced FDE. Moreover, lesions were detected in the oral cavity and in the perianal region. Small vesicles were apparent on top of some of the lesions along with some bullae, which later ruptured. The typical presentation of the well-demarcated oval red macules on the previous residual lesions and the appearance of new ones in unaffected skin supported the diagnosis of etoricoxib-induced FDE. Systemic symptoms were absent. The patient immediately discontinued etoricoxib and was advised to avoid etoricoxib and chemically related NSAIDs. Once again, the performance of a skin biopsy was proposed, but the patient did not consent. Due to the generalized distribution of the drug hypersensitivity reaction and the presence of vesicles and bullae on some macules, the patient was treated with methylprednisolone 0.5 mg/kg/day with gradual tapering, local antiseptic (0.1% octenidine hydrochloride 2%—phenoxyethanol *w*/*v* solution), and topical emollients. The erythema turned dusky, desquamated, and finally hyperpigmented during the next 4 weeks, while the sites of some lesions were still visible for almost 12 weeks. The performance of patch tests was proposed at least four weeks after the resolution of the eruption, but the patient did not consent. Despite that, the typical presentation of the well-demarcated oval red macules on some of the previous nimesulide-induced FDE lesions and the appearance of new macules in healthy skin after the use of etoricoxib, along with patient’s previous history of nimesulide-induced FDE, supported the diagnosis of FDE due to etoricoxib.

## 3. Review of the Literature

We conducted thorough literature searches on PubMed/Medline and Google Scholar, encompassing relevant studies up to and including November 2023. The goal was to identify and gather various types of publications (including retrospective studies, case series, and case reports) that documented patients developing fixed drug eruptions (FDE) or generalized bullous fixed drug eruptions (GBFDE) and exhibited cross-reactivity to non-steroidal anti-inflammatory drugs (NSAIDs), whether chemically related or unrelated. Using a combination of keywords related to the main study axes (NSAIDs, cross-reactivity, cross-reaction, intolerance, hypersensitivity reaction, FDE, GBFDE, cutaneous reaction, etc.) provided us with a comprehensive collection of relevant literature. Only publications in English were included, and the main characteristics of the studies and cases were extracted and summarized in Table 1 [10,11,12,13,14,15,16,17,18,19,20,21,22,23,24,25,26,27,28,29,30].

A total of three publications reported cross-reactivity among chemically unrelated NSAIDs [10,11,17]. Ammar et al. reported two female patients, aged 49 and 52, who developed multiple lesions typical of FDE after the ingestion of different NSAIDs. The initial workup consisted of patch tests that yielded negative results for both patients, but positive findings were observed with OPT. The first patient underwent OPT and had positive reactions for naproxen, indomethacin, ketoprofen, and tiaprofenic acid, and thus, the groups of propionic acid, fenamic acid, and indole derivates were contraindicated in this patient [10]. The second patient had positive OPT for lysine acetylsalicylate, mefenamic acid, and piroxicam and was treated accordingly [10]. Ouni et al. reported a case of a 40-year-old male that developed lesions 1 week after the initiation of mefenamic acid, paracetamol, and lansoprazole for post-traumatic knee pain. The patient experienced reactivation again after the ingestion of mefenamic acid. Since the role of mefenamic acid for the development of the cutaneous reaction was evident, the medication was withdrawn and testing for other NSAIDs was performed. Both patch tests and OPT were positive for diclofenac, and the patient was advised to abstain from those two NSAID groups [11]. Pérez-Calderon et al. reported an interesting case of a 52-year-old female patient who suffered from recurrent episodes of FDE due to the use of naproxen, while she had subsequently tolerated oral acetylsalicylic acid, paracetamol, and ibuprofen before medical consultation. She was subjected to patch testing with naproxen along with other NSAIDs without any positive results. Since it was important to identify safe alternatives to naproxen, oral challenge tests were performed and were positive for naproxen and nabumetone and negative for dexketoprofen. Notably, the patient had never used nabumetone before [17]. The role of patch testing in cases of unusual cross-reactivities may be limited, as supported by the presented cases, since it did not cause any reactions and OPT was needed to point out other potentially harmful medications that needed future avoidance [10,17].

All the other studies and cases retrieved report cross-reactivity reactions due to NSAIDs that are chemically related [12,13,14,15,16,18,19,20,21,22,23,24,25,26,27,28,29,30]. Despite that, such reactions still constitute the minority of the total cases of FDE and other cutaneous reactions caused by NSAIDs. A study involving 105 patients with confirmed FDE employed an OPT protocol to identify the offending agents. In this study, only one patient was reported to have a reaction to two medications of the same group, namely tenoxicam and piroxicam. In this study, OPT failed to identify the causative agent in 8 patients [20]. In addition, Kanwar et al. observed one positive reaction to phenylbutazone after OPT out of 6 patients with a positive reaction to oxyphenbutazone in a study with 98 FDE patients. The patients were subjected to OPT with the drugs that were taken at the time of the reaction as well as those that, by that time, were commonly known to cause fixed eruptions. Notably, in this analysis, the maximum number of FDE (50 cases; 51%) was due to trimethoprim-sulphamethoxazole followed by acetylsalicylic acid (24 cases; 24.5%) [25]. In a retrospective study, Andrade et al. evaluated 52 patients with FDE to assess the diagnostic value of patch testing in establishing an etiological diagnosis in FDEs. NSAIDs were the most common clinically suspected agents in 90.4% of the cases and were followed by antibiotics (28.9%), and paracetamol (15.4%). Patch tests on lesions were reactive in 21 patients (40.4%), and for 9 patients with positive test reactions to piroxicam, 8 were also reactive to tenoxicam and 2 to meloxicam, exhibiting high rates of cross-reactivity among those medications [15]. Interestingly, cross-reactivity between the oxicams was observed in many instances and included reactions among tenoxicam and piroxicam [15,18,20,21,22,24] or piroxicam and meloxicam [14,15,16]. In two patients, cross-reactivity was observed among piroxicam, tenoxicam, and droxicam. The first patient suffered from bullous FDE and had positive readings from his patch testing for those three substances, while the other, with only macular lesions, had negative patch testing for piroxicam but positive OPT for all the medications [22]. Two cases included reactions between the COX-2 inhibitors, etoricoxib, and celecoxib [12,13]. In the first patient, while etoricoxib was suspected initially, LTT was positive for etoricoxib, celecoxib, and sulfamethoxazole. Despite that, as implied by the authors, positive LTT to the agents apart from etoricoxib may not be of clinical relevance and can only highlight the possibility of a later reaction [12]. The second patient developed plaques and bullae after etoricoxib ingestion. Patch tests with etoricoxib and celecoxib were performed in both lesional and non-lesional skin (upper back), which induced new bullous lesions in the areas previously affected, along with negative reading on the back [12]. Other reactions reported in the literature included aceclofenac and diclofenac [19]. In this case, a 55-year-old woman developed an FDE lesion 10 h after the ingestion of aceclofenac, which resolved after 6–7 days without treatment. The patch tests with aceclofenac and diclofenac at 48 h and 96 h were positive on previously affected skin but negative on unaffected skin, while OPT for acetylsalicylic acid was negative [19].

Data from older studies have exhibited similar reactions among oxyphenbutazone and phenylbutazone [25,27,28,29,30], with the first drug now withdrawn from the markets due to its unacceptable safety profile and the later only used for special indications [31]. Similarly, cross-reactivity has been observed due to the use of phenazone and its derivatives [23,26], which are rarely used medications and, thus, do not constitute common real-life scenarios for physicians.

## 4. Discussion

FDE is a relatively common drug hypersensitivity reaction and its diagnosis in its typical form is usually straightforward due to lesion morphology and typical history of drug intake hours or days preceding the characteristic eruption [4]. To our knowledge, we present the first case of a nimesulide-induced FDE followed by etoricoxib-induced FDE in a patient with documented tolerance to etoricoxib, after the initial FDE reaction was attributed to nimesulide.

In the etoricoxib-induced FDE, the diagnosis primarily relied on clinical assessment. The clinical presentation unequivocally supported the diagnosis, as the ingestion of etoricoxib triggered the reactivation of previous lesions and the emergence of multiple new ones. Despite the occurrence of some bullous lesions that later ruptured (Figure 2C,D), the clinical presentation did not fulfill the proposed criteria for GBFDE [9]. Moreover, while a skin biopsy is indeed a valuable diagnostic tool and was advised in our case, it is not deemed indispensable for the diagnosis of FDE in its typical presentation. It is recommended when there is an atypical clinical presentation, the presence of systemic symptoms, or when the clinical diagnosis is uncertain, none of which were observed in our case [3,4].

As for nimesulide, positive patch tests and the corresponding clinical presentation confirmed the diagnosis of nimesulide-induced FDE. The pathomechanism behind FDE is not fully understood, but it is believed to be mediated by CD8+ memory T cells residing in the basal layer of the epidermis of FDE lesions. Upon exposure to the causative drug, these cells become reactivated, migrate upward in the epidermis, produce cytokines, and release cytotoxic molecules like granzyme B and perforin, leading to epidermal necrosis observed in FDE and its generalized form, GBFDE. Simultaneously, CD4+ regulatory T cells migrate into the lesions, resolving the local inflammatory process, contributing to the self-limited nature of FDE [32,33].

The presence of CD8+ memory cells in residual lesions justifies the positivity of patch tests when applied to these areas, with a sensitivity reaching up to 40% [34,35]. It is worth noting that LTT and OPT yielded negative results, with OPT being positive in other published cases. However, dosages and time intervals for OPT may differ among published studies, and it is uncertain if a single full therapeutic dose of the suspected causative agent is enough to induce hypersensitivity, as in our case [36,37,38]. In addition, LTT’s sensitivity and specificity vary in the literature, making it insufficient to definitively rule out hypersensitivity [39]. Moreover, the underlying pathomechanism may also explain the negative results of the LTT. LTT involves incubating a patient’s peripheral blood mononuclear cells (PBMCs) with the suspected culprit drug and measuring the proliferation rate compared to the patient’s unexposed PBMCs [12]. Although it can be useful in some patients to assess non-immediate hypersensitivity drug reactions, it is rarely employed to confirm FDE [40]. Moreover, although not commonly utilized in everyday clinical practice, one approach worth considering is pharmacogenetic testing, which assesses genetic variations that influence an individual’s response to medications. While this personalized approach to medication selection can potentially improve safety and efficacy by avoiding drugs that pose a higher risk of adverse reactions based on genetic predisposition, it is important to note that this method is not widely employed in clinical settings to prevent drug reactions, particularly FDEs [41].

Cross-reactivity between NSAIDs belonging in the same group and thus chemically related has been documented in the context of FDEs, as revealed by the search of the available published literature [12,13,14,15,16,18,19,20,21,22,23,24,25,26,27,28,29,30]. It should be noted that such reactions that are attributed to cross-reactivities between structurally related medications have been observed with the use of a variety of other agents including, but not limited to, quinolones, azoles, and antihistamines [4,42,43,44,45].

Based on the literature review, cross-reactivities among structurally unrelated NSAIDs have been reported, albeit infrequently. FDEs with cross-sensitivities among piroxicam, tenoxicam, and droxicam [22,24]; among piroxicam and meloxicam [14,15,16]; and among etoricoxib and celecoxib [12,13] have been reported in the literature, as well as with phenylbutazone and oxyphenbutazone [25,27,28,29,30]. On the other hand, cases similar to ours on FDE with cross-reactivity between chemically unrelated NSAIDs have been rarely reported [10,11,17]. Recently, Ammar et al. described two complex cases with unusual cross-reactivities. The first patient had an FDE with positive OPT for indomethacin, ketoprofen, and tiaprofenic acid, while the second had an FDE with positive OPT for lysine acetylsalicylate, piroxicam, and mefenamic acid with previous negative patch testing for those agents [10]. Another case by Ouni et al. reported that a patient with an FDE induced by mefenamic acid demonstrated cross-reactivity to diclofenac with positive patch testing and OPT [11]. Interestingly, Pérez-Calderon et al. described a patient with cross-reactivity among naproxen and nabumetone, which was only revealed after OPT with negative patch testing [17].

Given that etoricoxib was initially tolerated after the nimesulide-induced FDE, we can speculate that either more courses of etoricoxib were required to trigger the subsequent reaction by activating nimesulide-specific memory CD8 T cells or new etoricoxib-specific T cells were activated, indicating a new FDE reaction to a chemically unrelated NSAID, named etoricoxib.

It is important to note that while nimesulide and etoricoxib belong to different groups of NSAIDs and there have not been reports of cross-reactivity, both drugs are part of the non-antibiotic sulfa group. This means there is a theoretical possibility of cross-reactivity between them. However, there is no evidence in the literature supporting this idea. Even though they share similar reactive groups, cross-reactivity is not solely determined by their chemical structure; instead, it is mediated by the immune system, though the exact mechanisms are still unclear.

Sarkar et al. presented a case of nimesulide-induced fixed drug eruption (FDE) with potential cross-reactivity to co-trimoxazole FDE, speculated to be due to the common sulfa compound [46]. However, similar to how non-antibiotic sulfa drugs are generally well tolerated despite sulfa antibiotic allergies, cross-reactivity between various sulfonamide medications has not been backed by evidence, either structurally or clinically [47].

In fact, Storm et al. showed that penicillin was more likely to trigger allergic reactions than non-sulfa, non-antibiotic drugs among people allergic to sulfa antibiotics [48]. We believe that cross-reactivity to sulfonamide-containing drugs likely involves multiple concurrent hypersensitivity reactions, rather than being directly linked. This might be due to a higher predisposition to allergic reactions in general among patients allergic to certain drugs. However, this explanation does not fully account for the unusual case report we are discussing.

In addition, understanding the full clinical profile of patients experiencing adverse reactions such as health status, dietary habits, blood counts, hormonal and inflammatory markers, as well as liver and kidney function tests, could theoretically provide valuable insights into potential predisposing factors or underlying conditions contributing to the adverse reaction. By meticulously documenting and analyzing these clinical parameters alongside the occurrence of adverse events like FDE, clinicians can better understand the complex interplay of factors involved and potentially identify strategies for prevention or management in similar cases. However, it is essential to recognize that speculation regarding timing and biological mechanisms may still be limited by the current understanding of drug reactions and individual variability.

It is evident that our knowledge on the epidemiology, pathophysiology, and clinical manifestations of FDE has increased in recent years. Despite that, since in most cases, FDEs follow an indolent course, a possibly significant number of patients may not be assessed by specialists and rare cross-reactivities among medications might not be reported, warranting increased pharmacovigillance. Therefore, we cannot definitively exclude the possibility of immunological cross-reactivity between structurally unrelated NSAIDs, such as nimesulide and etoricoxib, or the development of new etoricoxib-specific T cells with no cross-reactivity in a susceptible patient. In either case, raising awareness of this clinical entity is crucial for both physicians and patients in the era of personalized medicine.

## 5. Conclusions

FDEs are relatively common among cutaneous drug reactions, frequently triggered by a variety of medications, with nonsteroidal anti-inflammatory drugs (NSAIDs) and antibiotics being the common culprits. While not very common, FDE can be caused by NSAIDs that share a common structure and chemical characteristics. As even chemically unrelated NSAIDs can be involved in FDE development, the present case and literature review underscore the need for increased awareness of the potential for cross-reactivity and the development of drug-specific T cells in the context of FDE. In the era of personalized medicine, a comprehensive understanding of these complex drug reactions is essential for both physicians and patients, emphasizing the importance of vigilant monitoring and thorough evaluation to ensure patient safety. Further research is warranted to shed more light on the mechanisms behind these intriguing drug eruptions and to improve diagnostic tools and management strategies.

## Figures and Tables

**Table 1:** Main characteristics of studies and cases that reported FDE with cross-reactivity among chemically related and unrelated NSAIDs.

Author, Year, Reference	Gender	Number of Patients (Male)	Age (Years)	Clinical Description of Initial Reaction	Affected Sites of Initial Reaction	Initial Suspected Agents	Interval Time from Agent Ingestion	Diagnostic Tests Performed	Cross-Reactivity (NSAIDs)
Ammar et al., 2022 [10]	0 Males 2 Females	2 (0)	Patient 1: 49 Patient 2: 52	Patient 1: three itchy erythematous plaques that faded spontaneously, leaving residual hyperpigmented patches Patient 2: four erythematous plaques	Patient 1: upper lip, chin, and right hand Patient 2: right and left wrists	Patient 1: mefenamic acid, naproxen, acetaminophen Patient 2: mefenamic acid, piroxicam	Patient 1: 1 day after (first episode) 1 day after (second episode) Hours after (third episode) Patient 2: 1 day after (all episodes before first visit)	Patient 1: after first and second episodes patch testing for naproxen (−) and acetaminophen (−) After third episode, skin biopsy indicative of FDE; patch test for naproxen (−); OPT for naproxen (+), indomethacin (+), ketoprofen (+), tiaprofenic acid (+), piroxicam (−), celecoxib (−), diclofenac (−), and acetaminophen (−) Patient 2: after first visit patch testing for lysine acetylsalicylate (−), mefenamic acid (−), piroxicam (−), naproxen (−), and celecoxib (−) OPT for lysine acetylsalicylate (+), mefenamic acid (+), piroxicam (+), naproxen (−), and celecoxib (−)	Patient 1: naproxen, indomethacin, ketoprofen, and tiaprofenic acid Patient 2: lysine acetylsalicylate, mefenamic acid, and piroxicam
Ouni et al., 2021 [11]	1 Male 0 Females	1 (1)	40	Multiple hyperpigmented lesions with pruritic erythematous and violaceous features	Upper extremities and trunk	Mefenamic acid, lansoprazole, paracetamol (first episode), mefenamic acid (second and third episode)	1 week (first episode), hours after (second episode)	Patch testing for diclofenac (+), celecoxib (−), and piroxicam (−), and OPT for diclofenac (+)	Mefenamic acid and diclofenac
Movsisyan et al., 2019 [12]	0 Males 1 Female	1 (0)	46	Pruritic maculopapular lesions on face and a single burning macular lesion twice at the same site on the left side of the abdomen	Face and abdomen	Etoricoxib	NA	LTT: etoricoxib (+), celecoxib (+), and sulfamethoxazole (+)	Etoricoxib and celecoxib
Carneiro-Leao et al., 2019 [13]	0 Males 1 Female	1 (0)	51	Multiple violaceous, pruritic, and painful plaques (shoulders and elbows); violaceous bullous lesion (right upper thigh) consistent with bullous FDE	Shoulders, elbows, and right upper thigh	Etoricoxib	NA	Patch testing for celecoxib (+) and etoricoxib (+)	Etoricoxib and celecoxib
Ben Romdhane et al., 2019 [14]	2 Males 5 Females	7 (2)	Mean: 40 (26–55)	Multiple (two to four lesions) in five patients and solitary lesions in two consistent with FDE. Bullous eruptions observed in two cases. Lesions were between 1 and 5 cm in size, were well demarcated, and left residual hyperpigmentation	The upper extremities were involved in five cases, followed by the face and the trunk	Piroxicam	2 days (2 hours to 3 days)	Patch testing for piroxicam (+) in 6/6 patients evaluated and meloxicam (+) in 1/7 patients evaluated, and OPT for meloxicam (+) for 2/6 patients evaluated	Piroxicam and meloxicam
Andrade et al., 2011 [15]	27 Males 25 Females	52 (27) (total patients)	Mean: 53 (20–78) (for all patients evaluated)	Lesions clinically consistent with FDE	NA	NSAIDs were suspected clinically in 47/52 cases, followed by antibiotics 15/52, paracetamol 8/52, and allopurinol 4/52.	NA	Patch testing (+) in 21/52 patients. Of these, 20 were reactive to NSAIDs including nimesulide (*n* = 9), piroxicam (*n* = 9), and etoricoxib (*n* = 2). For nine patients with patch testing positive for piroxicam, eight were also reactive to tenoxicam and two to meloxicam, whereas no reaction was observed to lornoxicam.	From nine patients with positive test reactions to piroxicam, eight were also reactive to tenoxicam and two to meloxicam
Ozdemir et al., 2011 [16]	0 Males 1 Female	1 (0)	44	Brownish, sharply demarcated and round pruritic plaques, and vulvar pruritus	Forearm, right knee, and vulvar region	Piroxicam, ibuprofen, and flurbiprofen	10–15 min	Patch testing for piroxicam (+), meloxicam (−), tenoxicam (−), and nimesulide (−) OPT meloxicam (+), naproxen (−), and nimesulide (−)	Piroxicam and meloxicam
Pérez-Calderon et al., 2011 [17]	0 Males 1 Female	1 (0)	52	Erythematous plaques with associated burning and itching sensation	Forehead and infraclavicular area	Naproxen	NA	Patch testing for naproxen (−), ibuprofen (−), ketoprofen (−), dexketoprofen (−), dexibuprofen (−), flurbiprofen (−), ketorolac (−), diclofenac (−), indomethacin (−), bufexamac (−), benzydamine (−), phenylbutazone (−), nabumetone (−), and piroxicam (−) OPT for naproxen (+), nabumetone (+), and dexketoprofen (−)	Naproxen and nabumetone
Ozkaya 2008 [18]	0 Males 1 Female	1 (0)	18	Circular indurated violaceous plaque, 10 mm in diameter	Left earlobe (sites of ear piercing)	Tenoxicam and piroxicam	Few hours	OPT for tenoxicam (+) and piroxicam (+)	Tenoxicam and piroxicam
Linares et al., 2007 [19]	0 Males 1 Female	1	55	Erythematous and pruritic lesion with residual hyperpigmentation	Back of the neck	Aceclofenac	10 h	Patch testing for aceclofenac (+) and diclofenac (+) OPT for acetylsalicylic acid (−)	Aceclofenac and diclofenac
Ozkaya-Bayazit 2003 [20]	53 Males 52 Females	105	Mean: 35.2 (4–67)	Lesions clinically consistent with FDE	Genital mucosa (50.5%), trunk (38.1%), lips (37.1%), and hands (32.4%) while other sites were affected less commonly	Trimethoprim-sulfamethoxazole (cotrimoxazole), naproxen, dipyrone, tenoxicam, piroxicam, paracetamol, and dimenhydrinate	NA	OPT trimethoprim-sulfamethoxazole (+) in 67 patients (63.8%), followed by naproxen (+) in 25 patients (23.8%), dipyrone (+) in 6 patients (5.7%), oxicam derivatives (+) in 5 patients (tenoxicam (+) in 3 and piroxicam (+) in 2) (4.8%), paracetamol (+) in 1 patient (0.95%), and dimenhydrinate (+) in 1 patient (0.95%). One patient reacted both to piroxicam and tenoxicam.	Tenoxicam and piroxicam in one patient
Oliveira et al., 1999 [21]	0 Males 1 Female	1	55	Erythematous, painful and edematous patches ranging from 2 to 8 cm	Trunk, buttocks, and thighs	Piroxicam	NA	Patch testing for piroxicam (+), tenoxicam (+), nabumetone (−), diclotenac (−), thiocolchicoside (−), antiglaucoma eye drops (−), NSAID series (−), and PCDRG series (−)	Piroxicam and tenoxicam
Ordoqui et al., 1995 [22]	1 Male 1 Female	2	Patient 1: 45 Patient 2: 40	Patient 1: Large edematous plaques with bullae Patient 2: Erythemato-violaceous macules	Patient 1: both elbows, distal hand phalanges, thighs, and the inguinal region Patient 2: left foot	Pt:1 piroxicam Pt:2 piroxicam	NA	Patient 1: Patch testing for piroxicam (+), tenoxicam (+), and droxicam (+) Patient 2: patch testing for piroxicam (−) and OPT for piroxicam (+), tenoxicam (+), and droxicam (+)	Patient 1: piroxicam, tenoxicam, and droxicam Patient 2: piroxicam, tenoxicam, and droxicam
Alanko 1994 [23]	18 Males 12 Females	30	16–76	Lesions clinically consistent with FDE	NA	16/30 phenazone salicylate	NA	Patch testing for phenazone salicylate (+) for all 16 patients Phenazone salicylate (per oral challenge performed in 3 out of 16 patients)	Cross-reactivity between phenazone derivatives was studied in eight patients with FDE caused by phenazone salicylate. Out of eight patients with a positive skin reaction to phenazone salicylate, eight showed a positive reaction to phenazone, three showed a positive reaction to aminophenazone, and four showed a positive reaction to propyphenazone.
Gastaminza 1993 [24]	0 Males 1 Female	1	57	Burning and itching on the upper extensor forearms and on the back; a circular erythema (2–3 cm in diameter)	Upper extensor forearms and back	Piroxicam	Next day (morning)	Patch testing for piroxicam (+), droxicam (+), tenoxicam (+), and thiosalicylic acid (−)	Piroxicam, droxicam, and tenoxicam
Kanwar et al., 1988 [25]	29 Males 69 Females	98	21–40 (3 patients less that 10 years old)	Lesions clinically consistent with FDE; 55 patients had a few lesions, 36 were classified as moderate, while there were 7 patients with GBFDE	Cutaneous involvement (43 patients), mucous membrane (33 patients), cutaneous with mucous membrane involvement (22 patients)	Trimethoprim-sulphamethoxazole (45), acetylsalicylic acid (24), hyoscine butylbromide (8), ibuprofen (6), oxyphenbutazone (6), tetracycline hydrochloride (2), phenolphthalein (1), and phenobarbitone (1)	NA	OPT for acetylsalicylic acid (+) in 24; ibuprofen (+) in 6; and oxyphenbutazone (+) in 6 patients, with 1 patient positive for phenylbutazone	Out of six patients with a positive reaction to oxyphenbutazone, one showed a positive reaction to phenylbutazone
Alanko et al., 1987 [26]	14 Males 10 Females	24	Mean 42.8 years, (21–71)	Lesions clinically consistent with FDE	NA	Phenazone salicylate	NA	Topical provocation testing for phenazone salicylate (+) in nine patients. Topical provocation with phenazone derivatives was performed in three patients, in which one FDE was originally caused by phenazone salicylate. A positive reaction to phenazone was obtained in all three patients. Provocation with propyphenazone and aminophenazone was observed in one patient. OPT in 2 of 9 patients with FDE due to phenazone salicylate and all 15 patients with FDE due to a drug other than phenazone salicylate had been challenged with the suspected drug and had given a positive reaction. One of the patients with a negative topical provocation test for propyphenazone and aminophenazone was later tested by oral provocation with the respective drugs with the results being negative.	In all three patients, a positive reaction was seen with phenazone, but only one patient showed positive results with propyphenazone and aminophenazone
Kanwar et al., 1984 [27]	M: F ratio = a little more than 2:1	106	21–40 (3 patients less that 10 years old)	Lesions clinically consistent with FDE	Patients with cutaneous involvement (44), mucous membrane involvement (23), and cutaneous and mucous membrane involvement (39)	Each patient was tested with the drugs commonly known to cause fixed eruptions as well as those being taken at the time of the reaction	NA	OPT was performed in 71 patients (not specified) (36 patients did not agree to provocation testing) Positive for acetylsalicylic acid (18), hyposcine butylbromide (15), oxyphenbutazone (14), sulphadiazine (7), tetracycline hydrochloride (6), metamizole (5), and ibuprofen (3)	Of 14 patients with a positive reaction to oxyphenbutazone, 6 patients were also tested with phenylbutazone, but only 2 showed a reaction
Pasricha 1979 [28]	NA	40	NA	Lesions clinically consistent with FDE	NA	Each patient was tested with the drugs commonly known to cause fixed eruptions and also those being taken at the time of the eruption	NA	Provocation test were not completed in 12 patients, and the causative drug could not be found. In the remaining patients, the causative drugs were shown to be tetracyclines (6), analgin (metamizole) (6), oxyphenbutazone (5), phenobarbitone (4), sulphadiazine (3), sulphaphenazole (2), penicillin (1), sulphadimethoxone (1), saridon (1), sulphadimidine (1), and sulphamethoxypyridazine (1)	Of five patients who showed a reaction with oxy-phenylbutazone, the one patient also tested with phenylbutazone showed evidence of cross-sensitivity
Pandhi and Dedi 1975 [29]	1 Male 0 Females	1	34	Round, erythematous and itchy patches on the back, arms and right palm; swelling of the lips	Back, arms, right palm, lips	Oxyphenbutazone	6 h	OPT testing for oxyphenbutazone (+) and phenylbutazone (+)	Oxyphenbutazone (+) and phenylbutazone (+)
Nayyar and Pasricha 1972 [30]	0 Males 1 Female	1	30	Dark red, oval, itchy patches	Arms, thighs, legs, lips	Oxyphenbutazone, sulphadiazine, vitamin B, codopyrin, and acetylsalicylic acid	Few hours	OPT testing for Sulfatriad (sulphadiazine, sulphamcrazinc and sulphadimidine), Crocin (paracetamol), Aspirin, and Codopyrine (Aspirin, Phcnacctin, and Codein phosphate) was negative. Positive OPT for oxyphenbutazone and phenylbutazone	Oxyphenbutazone (+) and phenylbutazone (+)

+: positive; −: negative; N/A: not applicable.

## Data Availability

The data that support the findings of this study are available from the corresponding author on request. The data are not publicly available due to privacy or ethical restrictions.
